# Effect of Inorganic Fillers on Electrical and Mechanical Properties of Ceramizable Silicone Rubber

**DOI:** 10.3390/polym16121695

**Published:** 2024-06-14

**Authors:** Mingyuan Yang, Jingqi Qiao, Bolin Su, Yongjian Xiao, Shenglin Kang, Yuchen Li, Hanzhong Cao, Hongchuan Tang, Xuetong Zhao

**Affiliations:** 1 State Key Laboratory of Power Transmission Equipment Technology, School of Electrical Engineering, Chongqing University, Chongqing 400044, China; 202211131207@stu.cqu.edu.cn (M.Y.); 202111131187@stu.cqu.edu.cn (J.Q.); 202111021022@stu.cqu.edu.cn (Y.X.); ksl124@cqu.edu.cn (S.K.); 202211131320@stu.cqu.edu.cn (H.C.); 2 Faculty of Electrical and Control Engineering, Liaoning Technical University, Fuxin 123032, China; su88364526@163.com; 3 State Grid Chongqing Electric Power Research Institute, Chongqing 401121, China; tanghongchuan1994@163.com

**Keywords:** ceramizable silicone rubber, inorganic fillers, ceramic samples, fire-resistant cable, electrical and mechanical properties

## Abstract

Ceramizable silicone rubber (CSR) composed of silicone rubber matrix and inorganic fillers can be transformed into a dense flame-retardant ceramic upon encountering high temperatures or flames. Conventionally, CSR can be sintered into a dense ceramic at temperatures above 1000 °C, which is higher than the melting point of a copper conductor used in a power cable. In this study, the vulcanization process and mass ratio of inorganic fillers of CSR were studied to lower its ceramization temperature to 950 °C. The electrical and mechanical properties of CSRs and their ceramic bulks were studied with various ratios of wollastonite and muscovite. It was found that the CSR samples could be successfully fabricated using a two-step vulcanization technique (at 120 °C and 150 °C, respectively). As a high ratio of muscovite filler was introduced into the CSR, the sample presented a high dc electrical resistivity of 6.713 × 10^14^ Ω·cm, and a low dielectric constant of 4.3 and dielectric loss of 0.025 at 50 Hz. After the thermal sintering (at 950 °C for 1 h) of the CSR sample with a high ratio of muscovite, the ceramic sample exhibits a dense microstructure without any pores. The ceramic also demonstrates excellent insulating properties, with a volume resistivity of 8.69 × 10^11^ Ω·cm, and a low dielectric loss of 0.01 at 50 Hz. Meanwhile, the three-point bending strength of the ceramic sample reaches a value of 110.03 MPa. This study provides a potential route to fabricate CSR used for fire-resistant cables.

## 1. Introduction

Fire-resistant materials were widely used in mineral-insulated cables, mica melt cables, and silicone rubber cables, which can be used to maintain the normal operation of power cables in the event of fire [1]. However, mineral-insulated cables and mica melt cables are susceptible to the environmental factors, such as moisture absorption and deformation, leading to insulation degradation and cable structure damage [2,3,4,5]. Conventionally, silicone rubber materials are developed through the copolymerization method to fabricate silicone-rubber-based precursors and doped with flame retardants, such as hydroxide, phosphorus-containing flame retardant, nitrogen-containing flame retardant, silicone-containing flame retardant, etc. [6,7,8,9], to achieve flame retardant properties. However, traditional flame retardant material will turn into powder once encountering a fire, thus losing its mechanical support [10].

Recently, ceramizable silicone rubbers (CSR) were developed as a novelty flame-resistant insulating material, and are composed of a silicone rubber matrix, fluxing agent, inorganic filler, and vulcanizing agent, exhibiting excellent thermal, electrical, and mechanical properties [11,12,13,14]. During the high-temperature combustion process, CSR can transform into robust ceramic materials on the surface of power cables to prevent the development of flames [15,16,17]. Hanu et al. [18,19,20] proposed that the ceramization process of CSR involves the melting of muscovite and a eutectic reaction with SiO_2_, resulting in a liquid phase that connects the filler and enhances the mechanical strength of the residual material. Mansouri et al. [21,22] showed that there was almost no sintering or eutectic reaction between the pyrolysis products and the filler at 600 °C, and the residual products present a low bending strength of only 0.3 MPa. As the temperature reached 1000 °C, muscovite began to melt and decomposed into SiO_2_ powder, which penetrated into the silicone rubber matrix and densified into a ceramic bulk with a strength of 3.2 MPa.

The mechanical properties of silicone rubber can be improved by establishing a well-linked filler network in the composite, which built an interface connection between the filler and silicone rubber. It was reported that the vulcanization process played a pivotal role in producing a well-organized filler network for the silicone rubber matrix without pores and agglomeration, which is a decisive factor for controlling the excellent mechanical properties of CSR and its ceramic bulk [20,21,22]. Moreover, low-melting-point fluxes were also crucial to improve the mechanical properties and flame retardancy of CSR composites and lower the densification temperature of ceramic bulk from CSR composites.

Moreover, Xu [23] demonstrated the feasibility of large-scale fireproof muscovite tape in fire-resistant cables. Chen [24] prepared silicon rubber (SIR) composites with four different fillers: aluminum hydroxide (ATH), montmorillonite (YMT), boron nitride (BN), and muscovite. The results show that the composites with BN and muscovite exhibited excellent insulating performances with a breakdown strength of 29.2 kV/mm and a failure rate of 5%, while the addition of YMT gave rise to a degraded insulating performance of SIR. Alexandrina et al. [25] studied the effect of different volume fractions of Al particles on the conductivity and dielectric polarization characteristics of silicone rubber composites. Nowadays, CSR as a potential flame retardant material was seldom studied for power cables, and the effect of dopants on its ceramization process is still unclear.

In this study, CSR composites were fabricated with various ratios of wollastonite and muscovite, which can be sintered into dense ceramic blocks at temperatures below the melting point of copper conductors, exhibiting excellent mechanical and electrical properties. Methyl vinyl silicone rubber was utilized as the matrix material, and wollastonite and muscovite were selected as the inorganic fillers. A two-step vulcanization technique (at 120 °C and 150 °C, respectively) was employed to fabricate CSR composites, and the relationships between the microstructure and the electrical and mechanical properties of CSR were analyzed. Afterwards, the CSR samples were sintered at 950 °C to obtain dense ceramic samples. The electrical, mechanical, and morphological characteristics of both CSR and the ceramic samples were tested and discussed. The results of this study can be applied to fire-resistant cable materials for insulation and flame retardancy.

## 2. Materials and Methods

### 2.1. Sample Preparation

The methyl vinyl silicone rubber was purchased from the Wacker-Chemie GmbH (Munich, Germany), which is composed of Si-O-Si main chains and a small amount (0.05 to 1 mol%) of vinyl on the side chains. The typical molecular structure is conducive to the vulcanization of raw rubber and improving the physicochemical properties of silicone rubber products [8,14]. The fluxing agent (glass powder with melting point of 450–550 °C) and vulcanizing agent (2,4-Dichlorobenzoyl peroxide (DCBP)) were both doped into the composite to enhance its temperature-resistant and antioxidative ability.

The wollastonite (CaSiO_3_) and muscovite (KAl_2_(AlSi_3_O_10_)(OH)_2_) were selected as inorganic fillers to improve the curing of CSR. These inorganic fillers with high hardness, strength and thermal stability can served as a supporting skeleton after the decomposition of the silicone rubber in the fire. Additionally, the muscovite (with melting point of 800 °C) [21,22] and fluxing agent with a low melting temperature will play a major role in the eutectic reaction with other residual decomposition of CSR. The liquid phase resulting from muscovite and the fluxing agent will promote the ceramization process of CSR at a low temperature of below 1000 °C. The raw materials and their suppliers are listed in Table 1.

CSR was prepared using a physical mixing method with different mass ratios of wollastonite and muscovite under the vulcanization temperature of 120 °C. Initially, methyl vinyl silicone rubber was placed in an open mill (model XH-401, Dongguan Xihua Machinery Technology Co., Ltd., Dongguan, China) with a gap of 1.5–2.5 mm between rolls. Subsequently, the glass powder, muscovite and DCBP were added sequentially into the silicone rubber. The open mill was running continuously at a speed of 15–20 revolutions per minute for one hour to obtain the composite material. Afterwards, the composite material was placed into a flat vulcanization machine (model CREE-6014A, Dongguan Kerui Instrument Technology Co., Ltd., Dongguan, China) in a stainless steel mold (dimensions: 100 mm × 100 mm × 3 mm) under a pressure of 10 MPa, and then vulcanized at 120 °C for 30 min. Finally, the composite material was transferred to an electric constant-temperature oven (model HZ-2014B, Dongguan Lyxian Instrument Technology Co., Ltd., Dongguan, China) for 5 h at 150 °C for the second vulcanization. The CSR samples with different mass ratios of wollastonite and muscovite were designated as R-40:0, R-30:10, R-20:20, R-10:30, R-0:40, respectively. The detailed formulations of CSR in this study are illustrated in Table 2.

**Figure 1:**
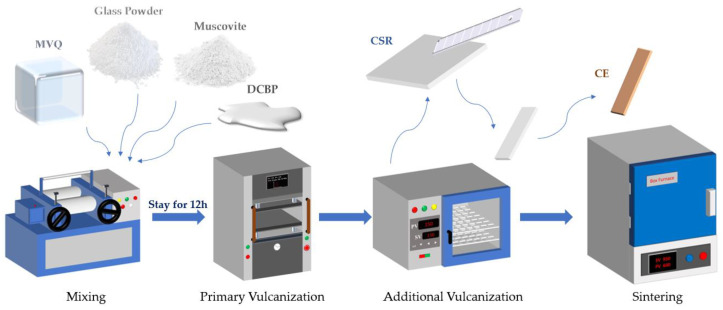
Ceramizable silicone rubber-based precursor vulcanization and sintering process.

To prepare the ceramic samples, CSRs were made into rectangle samples with a size of 80 mm × 10 mm × 3 mm. Subsequently, the rectangle samples were put into a corundum crucible covered with Al_2_O_3_ powder, and then sintered at 950 °C in a muffle furnace with a ramp rate of 2 °C/min. The samples were maintained at 950 °C for 1 h, and then furnace-cooled to room temperature. The vulcanization and sintering process is depicted in Figure 1. Finally, the ceramic samples are designated as C-40:0, C-30:10, C-20:20, C-10:30, and C-0:40, respectively.

### 2.2. Mechanical Performance Test

**Figure 2:**
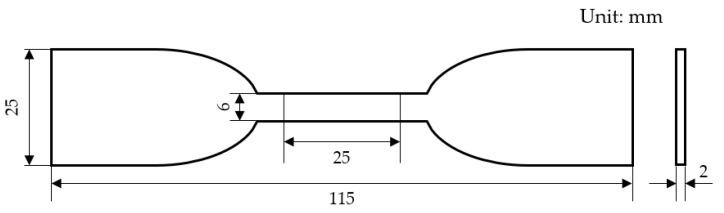
Geometry configuration and dimensions of the dumbbell-shaped sample designed for tensile test.

Dumbbell-shaped CSR samples were prepared and the elongation at break and tensile strength were tested using a WDW-100 model tensile testing device (Changchun New Testing Machine Co., Ltd., Changchun, China) with the tensile speed of 5 cm/s [26]. More than five dumbbell-shaped samples were tested to evaluate the measuring deviation. The geometrical dimension of the sample was shown in Figure 2.

The tensile strength is the equilibrium internal force per unit area that the material can withstand when subjected to an external force. It can be calculated using Equation (1).

(1) $$\sigma =\frac{F}{b\times d}$$

where *F* represents the maximum force exerted on the sample, and *b* and *d* denote the initial width (mm) and thickness (mm) of the sample, respectively. The elongation at break was calculated using Equation (2).

(2) $$\varepsilon =\frac{L-L_{0}}{L_{0}}\times 100\%$$

where *L*_0_ represents the initial distance (*L*_0_ = 20 mm), and *L* represents the distance (mm) between the marked lines on the sample and the point of break.

The flexural strength of the ceramic samples was assessed through the three-point bend method, employing the bending strength testing machine (model WBE-9000B, Guangdong WBE Scientific Research Instrument Co., Ltd., Dongguan, China). The samples with dimensions of 8 mm in width and 3 mm in thickness were used for the three-point bending strength test, and the bending strength was calculated using Equation (3).

(3) $$S=\frac{3PH}{2tk^{2}}$$

where *H*, *k*, *t* and *P* are the initial distances set by the machine (*H* = 20 mm), the thickness of the sample (*k* = 3 mm), the width of the sample (*t* = 8 mm), and the maximum load (N), respectively.

### 2.3. Electrical Performance Test

The dielectric properties of CSR and the sintered ceramic samples were measured in the frequency range from 10^0^ to 10^6^ Hz using the broadband dielectric spectrometer (Novocontrol, Concept 80, Montabaur, Germany). The volume resistivity of the CSR samples was tested with the megger (model 6517B, Keithley Company, Beaverton, OR, USA) at a voltage of 1000 V in accordance with GB/T 1692-2008. The volume resistivity of the ceramic samples was calculated using the four-point probe tester (FP-001, Zhuhai Kaivo Instruments and Equipment Co., Ltd., Zhuhai, China).

### 2.4. Calculation of Shrinkage Rate

The shrinkage rate of CSR after sintering was measured using Equation (4).

(4) $$R=\frac{r_{1}-r_{2}}{r_{1}}\times 100\%$$

where *R* is the linear shrinkage (%), *r*_1_ is the length of CSR (mm), and *r*_2_ is the length of ceramic bodies (mm) after sintering.

### 2.5. Density of Ceramic Samples

The density of ceramic sample was calculated using the Archimedes method based on Equation (5):

(5) $$\rho =\frac{m_{0}}{m_{1}-m_{2}}\times \rho_{w}$$

where *m*_0_ represents the mass of the desiccated ceramic sample (g), *m*_1_ signifies the saturated wet weight of the ceramic (g), *m*_2_ denotes the weight of the ceramic immersed in anhydrous ethanol (g), and *ρ*_w_ is the density of anhydrous ethanol (g·cm^−3^).

### 2.6. Microstructure Observation

The cross-sectional surfaces of ceramic samples were sputter-coated with gold by the sputter coater (Model EMS 150R S, Quorum Technologies Ltd., Laughton, UK) to observe their microstructural morphology using a field emission scanning electron microscope (FESEM) (300 VP SEM, Zeiss Gemini Sigma, Oberkochen, Germany).

### 2.7. Error Analysis

The error bars were calculated with the experimental standard deviation using Equation (6).

(6) $$s=\sqrt{\frac{\sum_{i=1}^{n}{\left(X_{i}-\bar{X}\right)}^{2}}{n-1}}$$

where *s* is the experimental standard deviation, *X*_i_ is the measured values, $\overset{-}{X}$ is the arithmetic mean value, and *n* is the number of repeated measurements.

## 3. Results and Discussion

### 3.1. Effect of Inorganic Fillers on the Properties of CSR

Silicone rubber is a weak polar dielectric material, and its conductivity is largely determined by the impurity of ions [27]. These impurities may originate from trace amounts of sulfide products generated during the vulcanization process, residual small molecules, and fillers. Under an external electric field, impurities may ionize and result in enhanced ionic conductivity.

**Figure 3:**
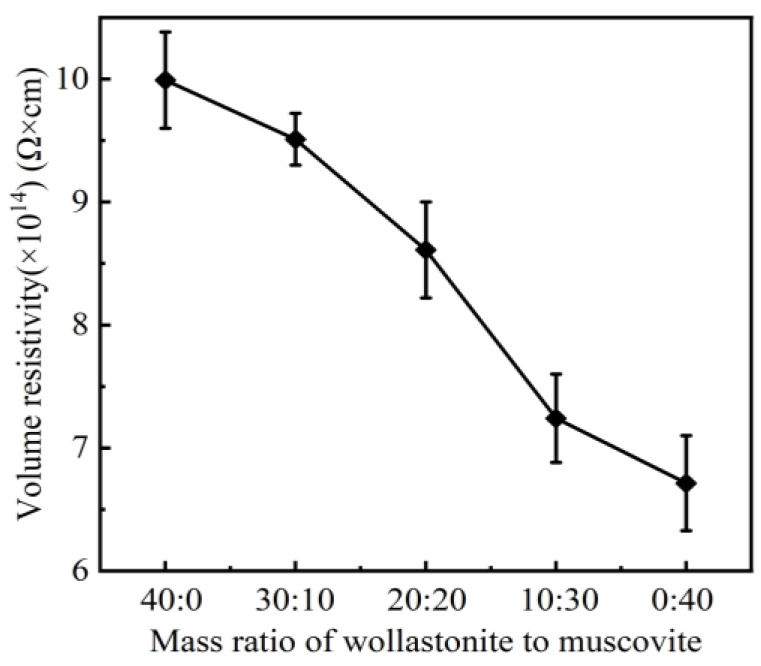
Volume resistivity of CSR with different mass ratio of inorganic fillers.

The effects of wollastonite and muscovite on the electrical properties of the silicone rubber are shown in Figure 3. At a ratio of 40:0, the volume resistivity of CSR reaches 9.99 × 10^14^ Ω·cm. As the amount of muscovite increases, the volume resistivity of CSR gradually decreases to 6.713 × 10^14^ Ω·cm. Muscovite is composed of SiO_2_ and a variety of metal oxides (MgO, K_2_O, Al_2_O_3_, CaO) [28,29]. Under the action of an electric field, metal ions are ionized and the concentration of carriers in CSR increases, resulting in a decrease in volume resistivity [30,31]. Meanwhile, doped, layered muscovites are difficult to homogeneously disperse in the silicone rubber matrix, and the agglomerated particles may induce interface defects in CSR, provide an electrical conduction path, and give rise to a decreased volume resistivity [32]. Therefore, as the muscovite content increases, the volume resistivity of CSR is reduced, as shown in Figure 3. However, it still meets the requirement that the volume resistivity of insulating materials is no less than 1.0 × 10^11^ Ω·cm [33,34,35].

**Figure 4:**
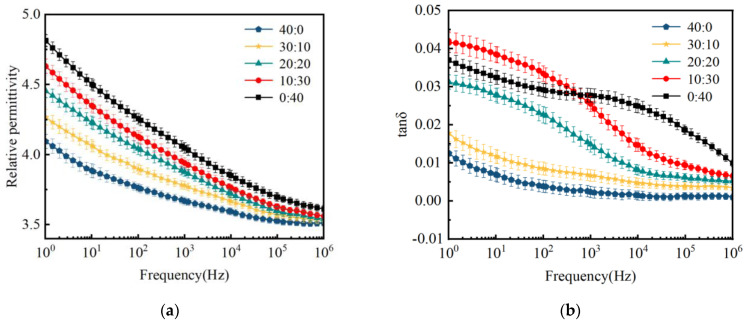
Dielectric property of CSR with different mass ratios of inorganic fillers: (**a**) dielectric constant; (**b**) dielectric loss.

Figure 4 shows the variation in the dielectric constant (*ε*_r_) and dielectric loss (tan*δ*) for CSR samples with different mass ratio of inorganic fillers. With the increase in muscovite content, the dielectric constant of the CSR composites gradually increases, while the dielectric loss exhibits a trend of initially increasing and then decreasing. The polymer composites with various fillers consist of three phases: the matrix phase (polymer), the dispersed phase (fillers), and the interfacial phase (the interface region between the polymer and fillers) [36]. In most cases, the interfacial phase plays a decisive role in the dielectric performance of composite materials. Muscovite, as a layered mineral composed of silicate layers and metal ion layers, exhibits ionization under the role of an external electric field, which may lead to interface polarization effects, elevating the dielectric constant.

Figure 4b demonstrates that the dielectric loss of the composite materials shows a decreasing trend with increasing muscovite content because wollastonite has a smaller density and is difficult to uniformly disperse in the silicone rubber matrix, which results in a more compact molecular structure, thereby constraining carrier mobility and diminishing the impact of leakage conductance on dielectric loss [37,38]. Moreover, the tendency for filler agglomeration reduces the free volume of the silicone rubber matrix, restricting the orientation polarization of dipoles in the composite material.

**Figure 5:**
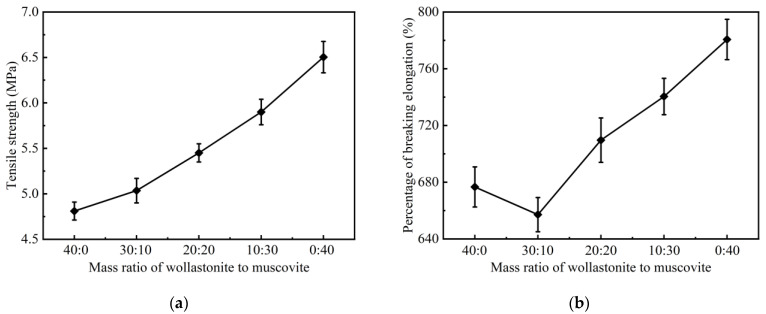
Mechanical properties of CSR with different mass ratios of ceramic inorganic fillers: (**a**) tensile strength; (**b**) percentage of breaking elongation.

Figure 5 illustrates the influence of different mass ratios of wollastonite to muscovite on the tensile strength and fracture strain of CSR samples. With the increase in muscovite proportion, the tensile strength of the CSR increases from 4.81 MPa to 6.50 MPa, while the elongation at break decreases to a local minimum of around 657.11% at a mass ratio of 30:10 (wollastonite to muscovite), and then increases to 780.60%, which can be attributed to the smaller particle size of muscovite. When the wollastonite is added into silicone rubber, the sponge-like structure with an amount of voids will be produced in the composites [39,40]. This typical structure can lead to a high elongation at break but a low tensile strength of composites [41,42,43] since muscovite was introduced to improve the polymer-filler interface due to its good compatibility with the silicone rubber matrix [44,45,46]. When wollastonite and a small amount of muscovite (30:10) are both added to the silicone rubber, the sponge-like structure may be damaged and give rise to a high hardness of the composite [47], resulting in a reduced elongation at break. Moreover, Skelhorn et al. reported that a strong adhesion between filler particles and the polymer matrix would be achieved as more muscovite was added [48], which can increase the elongation at break due to the typical layered structure and sliding capabilities of muscovite [38,49,50]. Therefore, as the mass ratio of muscovite to wollastonite increases, a higher elongation at break of the composited was obtained.

### 3.2. Effect of Inorganic Fillers on the Morphology of Ceramic Samples

**Figure 6:**
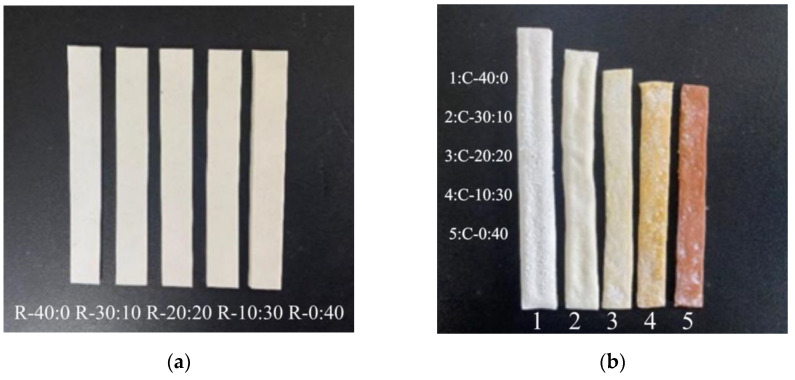
Surface morphology of samples with different mass ratios of ceramic inorganic fillers, (**a**) CSR and (**b**) ceramic samples.

As shown in Figure 6a, the silicone rubber samples were cut into strips with dimensions of 80 mm × 10 mm × 3 mm. Then, the samples were sintered at 950 °C to obtain ceramic bulks as presented in Figure 6b. The ceramic samples maintain the overall shape and contour of the CSR samples. Specifically, the ceramic sample (C-40:0) without doping muscovite has a white color and exhibits an uneven surface with clear warping. However, as the muscovite content increases, the surface warping decreases and becomes smooth. Additionally, the color of the samples gradually changes from the white of sample C-40:0 to the brownish-yellow of sample C-0:40.

**Figure 7:**
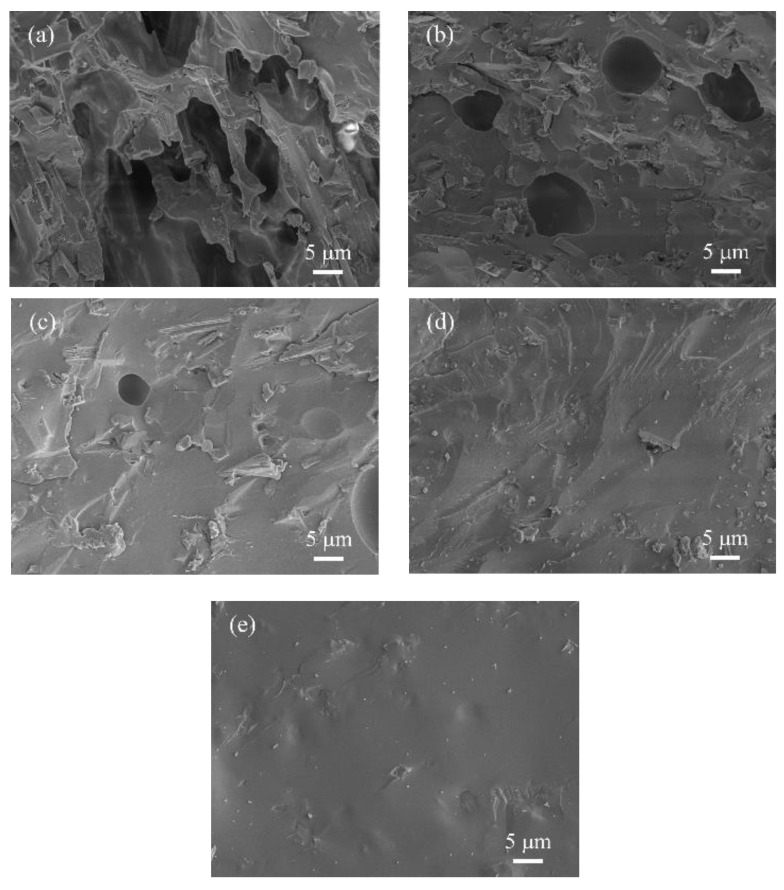
The cross-sectional morphology of ceramic samples with different mass ratios of inorganic fillers (wollastonite to muscovite): (**a**) 40:0; (**b**) 30:10; (**c**) 20:20; (**d**) 10:30; and (**e**) 0:40.

During the sintering process of CSR, the silicone rubber matrix is the first to decompose into small molecules and partially volatilize. As the temperature further rises to the melting point of glass powder (450–550 °C), it melts to the liquid phase; meanwhile, the silicone rubber matrix completely decomposes into SiO_2_. Afterwards, a eutectic reaction takes place among the muscovite, the residual SiO_2_, and the liquid phase, promoting the ceramization of the mixture when the temperature reaches 950 °C. The cross-sectional microstructure of the ceramic samples was obtained by SEM. Figure 7 shows the cross-sectional microstructure of ceramic samples made from CSR with various mass ratios of wollastonite and muscovite. During ceramic sintering, the glass powder would melt into the liquid phase at high temperatures, connecting the filler particles, while also promoting bonding between the particles. During the cooling process, the liquid phase solidifies, forming a dense structure between particles, thereby increasing the density of the ceramic material. It can be found that the liquid phase was produced by the melt of the glass powder mixed with the filler particles, inducing a complete “bridge” structure [51].

As shown in Figure 7a,b, the ceramic samples with a high proportion of wollastonite (C-40:0, C-30:10) exhibited a porous and layered structure. The cross-section structure showed a large amount of defects, such as layer stacking and pores. As the muscovite content increases, the number of defects decreased (C-20:20, C-10:30, C-0:40), and the distribution of the liquid phase and filler particles became more homogeneous, resulting in a smooth and dense structure, which can be attributed to the smaller particle size of muscovite compared to wollastonite and better dispersion of muscovite in the silicone rubber matrix [52]. Moreover, the melting point of muscovite is smaller than wollastonite, and they can be mixed with the liquid phase to form a compact eutectic at 950 °C. Figure 7e shows that the internal microstructure of ceramic sample is dense without any pores, which can isolate air and block the heat transfer from outside. It can prevent combustion and suppress the thermal decomposition of silicone rubber, thereby exerting a fire retardant effect. Meanwhile, it was reported that the layered structure of muscovite would also slow down the rate of heat transfer [53,54].

### 3.3. Effect of Inorganic Fillers on Ceramic Samples

**Figure 8:**
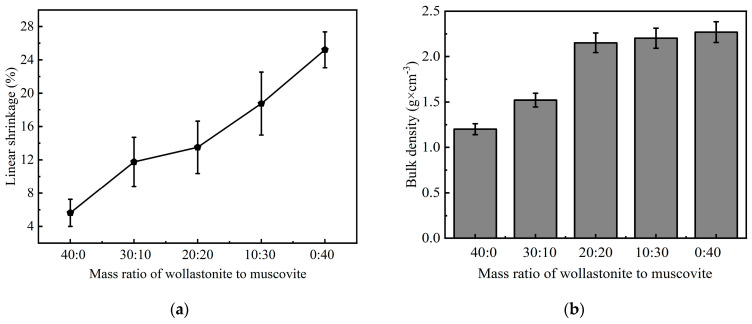
Linear shrinkage and density of ceramic samples with different mass ratios of inorganic fillers, (**a**) linear shrinkage, and (**b**) bulk density.

The linear shrinkage of the CSR after sintering is shown in Figure 8a. It can be observed that with the increase in the proportion of muscovite, the ceramic samples exhibit a greater shrinkage, reaching the maximum value of 25.21% for the sample C-0:40. It is believed that this is caused by the decomposition of silicone rubber during the sintering process and the reduction in the number of pores within the ceramic sample. Furthermore, muscovite creates cross-linking networks, which greatly suppress the outward diffusion of small molecules [55]. Moreover, Gupta et al. reported that cross-linking networks could prevent adjacent chains from moving too far away from one another, inducing a high cross-linking density which could be reflected by its high shrinkage ratio during the ceramization process at high temperatures [56]. Meanwhile, the liquid phase generated by the glass powder during sintering can mix with muscovite and fill the pores, thus contributing to a distinct shrinkage of ceramic samples and reaching a high density of 2.268 g/cm^3^, as shown in Figure 8b. As a result, the ceramic samples exhibit a dense internal microstructure, as shown in Figure 7.

**Figure 9:**
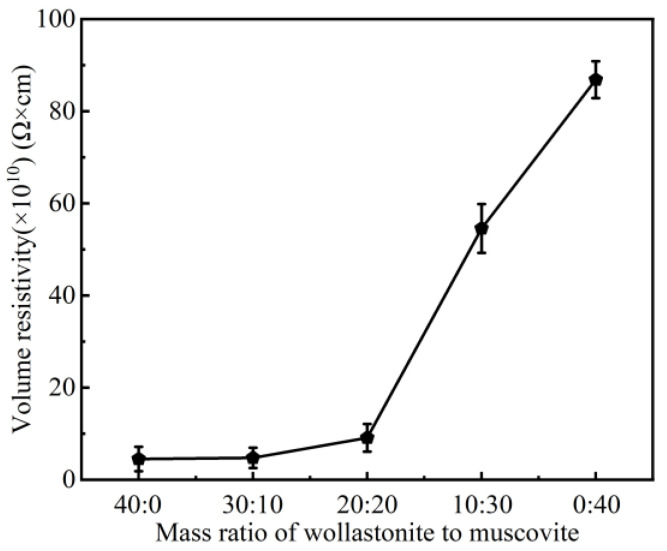
Volume resistivity of ceramic samples with different mass ratio of inorganic fillers.

In Figure 9, the volume resistivity of the ceramic samples was calculated according to the volume resistance tested with the megger (model 6517B). It can be found that the volume resistivity of the ceramic samples increased with the increase in muscovite content, indicating a decrease in dc conductivity. As the mass ratio changed from 20:20 to 0:40, there was a sharp increase in volume resistivity. The volume resistivity of the composite dramatically increased from 8.80 × 10^10^ Ω·cm to 8.69 × 10^11^ Ω·cm, indicating the presence of a percolation transition around the 20:20 mass ratio [57,58,59]. At this mass ratio, the segregated conductive network in the composite was produced, resulting in a significant increase in volume resistivity. It is believed that the addition of muscovite promotes a dense internal structure with fewer pores and stronger adhesion among components, leading to the obstruction of conductive pathways and high volume resistivity [60,61].

The classical Debye theory effectively describes a single relaxation phenomenon in a dielectric constant, neglecting the influence of direct current (dc) conductivity (*γ*). The Debye equation can be expressed as Equation (7) [62].

(7) $$\begin{array}{l} \varepsilon^{*}=\varepsilon^{\prime }-i\varepsilon^{\prime \prime }=\varepsilon_{\infty }+\frac{\varepsilon_{s}-\varepsilon_{\infty }}{1+i\omega \tau }\\ =\varepsilon_{\infty }+\frac{\varepsilon_{s}-\varepsilon_{\infty }}{1+\omega^{2}\tau^{2}}-i\frac{(\varepsilon_{s}-\varepsilon_{\infty })\omega \tau }{1+\omega^{2}\tau^{2}} \end{array}$$

where ε* represents the complex dielectric constant; *ε*′, *ε*″ are the real part and imaginary part of the complex dielectric constant, respectively; *ε_s_* and *ε*_∞_ are the static and optical dielectric constants, respectively; *ω* is the angular frequency; and *τ* is the relaxation time. The imaginary part of the complex dielectric constant *ε*″ is proportional to the active current value of the dielectric constant, known as the loss factor. For the ceramic samples, the influence of dc conductivity *γ* is often significant, especially in the low-frequency region. When deriving the formula for dielectric loss, the role of dc conductivity *γ* needs to be considered. Thus, the loss factor can be expressed as Equation (8) [62,63]:

(8) $$\varepsilon^{\prime \prime }=\frac{\gamma +g}{\varepsilon_{0}\omega }$$

(9) $$g(\omega )=\varepsilon_{0}\omega \varepsilon^{\prime \prime }=\frac{\varepsilon_{0}(\varepsilon_{S}-\varepsilon_{\infty })\omega^{2}\tau }{1+{\left(\omega \tau \right)}^{2}}$$

where *ε_s_* is the dielectric constant of vacuum, *g* represents the equivalent alternating current conductivity of the dielectric, which is closely related to the relaxation process. Therefore, tan*δ* can be expressed by Equation (10):

(10) $$\tan\delta =\frac{\varepsilon^{\prime \prime }}{\varepsilon^{\prime }}=\frac{\gamma +g}{\varepsilon_{0}\varepsilon_{r}\omega }$$

where *ε_r_* is the relative dielectric constant of the dielectric.

**Figure 10:**
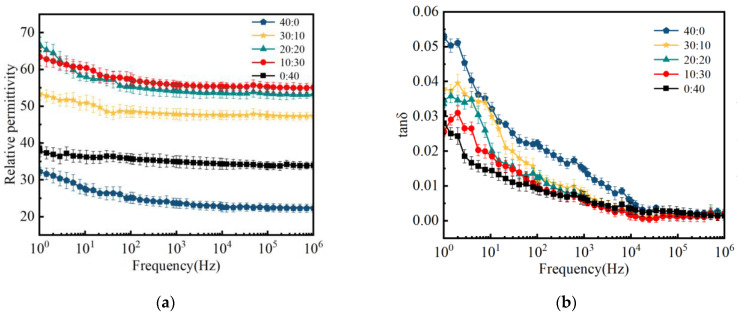
Dielectric property of ceramic samples with different mass ratio of inorganic fillers: (**a**) dielectric constant and (**b**) dielectric loss.

Figure 10 shows the dielectric properties of ceramic samples as a function of frequency. The results indicate that the dielectric constant of the ceramic samples remains relatively stable with increasing frequencies. As the mass ratio of wollastonite to muscovite varied from 40:0 to 10:30, the dielectric constant (at 50 Hz) of the ceramic samples increased from 24.27 to 57.78. However, when the ratio continues to increase to 0:40, the *ε_r_* of the ceramic sample significantly decreases to 0.01. Moreover, the dielectric loss of the ceramic sample shows a downward trend with increasing muscovite content, especially at low frequencies. According to Equation (9), the decreasing electrical conductivity (the reciprocal of the volume resistivity) of the ceramic samples (as shown in Figure 9) also plays a great role in restraining the dielectric loss at low frequencies, as shown in Figure 10b [64]. Moreover, a small loss peak at low frequencies might be a result of the equivalent alternating current conductivity *g*, which is related to the impurity ion polarization caused by the doped fillers or glass powder.

**Figure 11:**
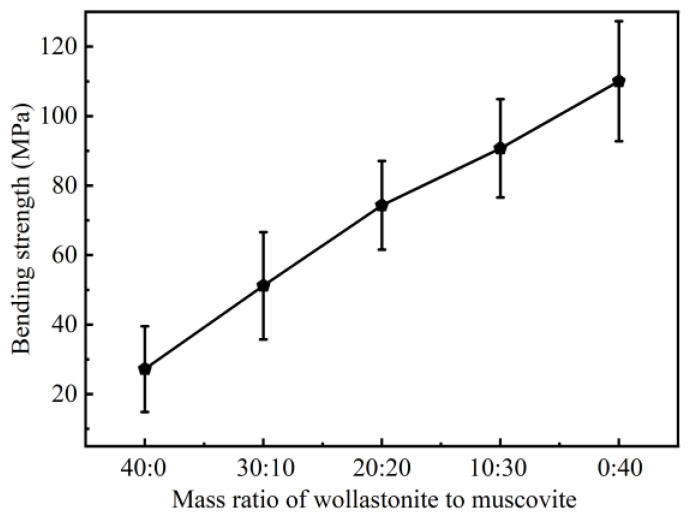
Three-point bending strength of ceramic samples with different mass ratios of inorganic fillers.

The three-point bending strength of ceramic samples with different mass ratios of inorganic fillers is presented in Figure 11, indicating that the increase in muscovite content improves the three-point bending strength. When muscovite is used as the sole inorganic filler (0:40), the three-point bending strength reaches 110.03 MPa, about five times higher than that when wollastonite is used as the sole inorganic filler (40:0). This value is also notably superior to the range of 14–61 MPa reported in previous studies [26,55,65,66]. In Figure 7, SEM results reveal that the internal structure of the ceramic sample exhibits numerous pores when only wollastonite is used as the inorganic filler, leading to a low mechanical strength. On the other hand, muscovite plays a positive role in reinforcing the mechanical strength of the ceramic samples, as it builds a continuous structure with the aid of fluxing agents and enhanced muscovite adhesion within the matrix [67]. Consequently, the ceramic sample with a 0:40 ratio exhibits a high three-point bending strength.

## 4. Conclusions

The effects of different mass ratios of inorganic fillers on the properties of CSR and its ceramic samples were investigated in this study. It was found that the increasing concentration of muscovite during the vulcanization process at 120 °C had a significant effect on improving the electrical and mechanical properties of CSR. The sample R-0:40 containing 100% muscovite as the optimal recipe exhibits a high resistivity of 6.713 × 10^14^ Ω·cm, a low dielectric constant of 4.3, and a dielectric loss of 0.025 at 50 Hz, respectively. The overall tensile strength can be enhanced by 20.9%. The ceramic sample C-0:40 sintered from sample R-0:40 at 950 °C shows a dense internal structure and a high three-point bending strength of 110.03 MPa, which enables its potential application in fire-resistant cables.

## Figures and Tables

**Table 1:** Raw materials and their suppliers.

Raw Materials	Suppliers
Methyl Vinyl Silicone Rubber	Wacker-Chemie GmbH (Munich, Germany)
Glass Powder	Lianyungang Oawa Materials Technology Co., Ltd. (Lianyungang, China)
Wollastonite	Jiangxi Kete Fine Powder Co., Ltd. (Yichun, China)
Muscovite	Jiangxi Aote Technology Co., Ltd. (Yichun, China)
2,4-Dichloro-Benzoyl Peroxide (DCBP)	Dongguan Cai Meijia Electronic Technology Co., Ltd. (Dongguan, China)

**Table 2:** Experimental formulations of the CSR samples.

Formulation	Content/g
Methyl Vinyl Silicone Rubber	100
Glass Powder	30
Wollastonite	40/30/20/10/0
Muscovite	0/10/20/30/40
2,4-Dichloro-Benzoyl Peroxide (DCBP)	2

## Data Availability

The data presented in this study are available in article.
